# Daily motionless activities: A dataset with accelerometer, magnetometer, gyroscope, environment, and GPS data

**DOI:** 10.1038/s41597-022-01213-9

**Published:** 2022-03-25

**Authors:** Ivan Miguel Pires, Nuno M. Garcia, Eftim Zdravevski, Petre Lameski

**Affiliations:** 1 Instituto de Telecomunicações, Universidade da Beira Interior, 6200-001 Covilhã, Portugal; 2 Escola de Ciências e Tecnologia, University of Trás-os-Montes e Alto Douro, Quinta de Prados, 5001-801 Vila Real, Portugal; 3 Faculty of Computer Science and Engineering, University Ss Cyril and Methodius, 1000 Skopje, North Macedonia

**Keywords:** Computer science, Data acquisition, Quality of life

## Abstract

The dataset presented in this paper presents a dataset related to three motionless activities, including driving, watching TV, and sleeping. During these activities, the mobile device may be positioned in different locations, including the pants pockets, in a wristband, over the bedside table, on a table, inside the car, or on other furniture, for the acquisition of accelerometer, magnetometer, gyroscope, GPS, and microphone data. The data was collected by 25 individuals (15 men and 10 women) in different environments in Covilhã and Fundão municipalities (Portugal). The dataset includes the sensors’ captures related to a minimum of 2000 captures for each motionless activity, which corresponds to 2.8 h (approximately) for each one. This dataset includes 8.4 h (approximately) of captures for further analysis with data processing techniques, and machine learning methods. It will be useful for the complementary creation of a robust method for the identification of these type of activities.

## Abstract (summary)

**Table:** 

Measurement(s)	motion sensors • GPS navigation system • Microphone Device • physical activity
Technology Type(s)	Smartphone Application • Artificial Intelligence
Factor Type(s)	raw data
Sample Characteristic - Organism	Individual Behavior
Sample Characteristic - Environment	road • house
Sample Characteristic - Location	Municipality of Fundao • Municipality of Covilha

## Background & Summary

Human activity recognition (HAR) has been one of the most challenging and at the same time most popular problems for scientific research. There are many published datasets that allow researchers to experiment and evaluate their approaches that tackle this problem. For example, The Human Activity Recognition Using Smartphones Dataset^1^, popularly known as UCI-HAR dataset has been used in many research publications. This dataset has three motion and three stationary activities recorded using the smartphone embedded sensors. Another popular dataset is the WISDM^2^ dataset, that consists of similar activities recorded using a Smartphone. The SHL^3^ dataset is one of the more recent datasets that uses smartphone sensors for HAR during transport. The University of Dhaka (DU) Mobility Dataset (MD)^4^ is another available dataset that uses wearable sensors for activities of daily living detection. Most of the available datasets combine both motionless and motion activities or focus on motion activities and falls. Also, the Human Activity Recognition Trondheim dataset (HARTH)^5^ is another dataset composed by accelerometer data related that combines several activities recorded during free living. The ExtraSensory^6^ dataset contains a large dataset with several activities, including motion and motionless activities, composed by a lot of sensors, including accelerometer, gyroscope, magnetometer, watch accelerometer, watch compass, location, audio, audio magnitude, and others. Finally, containing data acquired from sensors available in smartphones and smartwatches, there are a lot of datasets available in CrowdSignals.io containing motion and motionless activities’ data, e.g., the AlgoSnap^7^ dataset.

The available datasets in the literature are mostly focused on combination of motionless and motion activities. The dataset presented in this paper focuses on motionless activities, especially when the person involved does very little or no motion at all during the activity. This dataset would allow scientist to focus on such activities that are usually hard for algorithms and models to distinguish when combined with motion activities.

Along the time, several researchers have been studied the identification of motionless activities with the sensors available in mobile devices^8–10^ for further application the different scenes related to Ambient Assisted Living and Enhance Living Environments. The presented data intends to present inertial, acoustic, and location data for further integration to create an automated system for the personalized monitoring of lifestyles. These data were collected with different people with distinct lifestyles and location for further generalization of the results obtained with this dataset to create a reliable system for the recognition of motionless activities^11^.

The dataset presented in this paper includes various sensors, including accelerometer, gyroscope, magnetometer, microphone, and GPS sensors. The data was collection during three motionless activities, including sleeping, driving, and watching TV. The data was acquired with a BQ Aquaris 5.7 smartphone^12^, including the pants pockets, in a wristband, over the bedside table, on a table, inside the car, or on other furniture.

The data was collected by 25 individuals (15 men and 10 women) in different environments around Covilhã and Fundão municipalities (Portugal). The data related to the different sensors was acquired with a sampling rate of 100 Hz by the accelerometer sensor, 50 Hz by the magnetometer sensor, and 100 Hz by the gyroscope sensor. Also, the used GPS receiver integrates an advanced dual frequency GNSS receiver with a 28 nm CMOS dual processor, reporting frequencies between 10.23 MHz for GPS L5, and 1.023 MHz for GPS L1. The sample of the microphone data is 44100 Hz collected into an array with 16-bit unsigned integer values in the range [0, 255] with a 128 offset for zero.

**Fig. 1:**
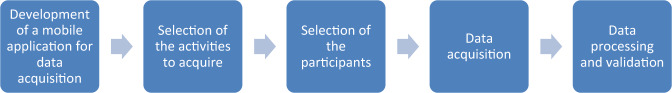
Workflow of the dataset creation.

The study that included the use of this dataset consists of the identification of Activities of Daily Living and environment with the data acquired for a commonly used mobile device. Thus, Fig. 1 presents the structure of the study for the data acquisition and processing.

This dataset is important to different kinds of people for different reasons. These are:

- The presented dataset allows the implementation of techniques to automatically identify the proposed motionless activities for the increasing functionality of the recognition of activities with motion detectable. It includes common motionless activities performed by a major part of the people;
- The data will allow the development of automatic methods for the identification of the proposed motionless activities, and the promotion of the increasing physical practice;^13,14^
- The use of mobile devices for the data acquisition, integrating the acquisition of acoustic, location, and inertial data allows the identification of motionless activities, which complement the creation of a Personal Digital Life Coach;^15^
- It allows the people’s monitoring during motionless activities, allowing the identification of possible accident, which may occur everywhere;
- Big data and machine learning techniques are important to allow the mentoring of some activities and environments^16^. These data represent the combination of several types of sensors and data that allows the development of complex and multivariate solutions for the monitoring of activities and environments.

## Methods

### Participants

The data acquisition was performed twenty-five volunteering individuals (15 men and 10 women) aged between 16 and 60 years old (33.5200 ± 13.5250 years old). All participants provided written informed consent before the experiments to allow us to share the results of the tests in an anonymous form. The agreement also provided the participants’ informed consent considering the risks and the objective of the study. Only the data related to the individuals that sign the consent to participate in the study were recorded. The participants were also informed that about the inclusion of the data anonymously in Mendeley Data. Ethics Committee from Universidade da Beira Interior approved the study with the number CE-UBI-Pj-2020–035. Due to the proximity to our research center, the data acquisition was performed in different environments in Covilhã and Fundão municipalities (Portugal). As it is included in a project related to the identification of Activities of Daily Living (ADL), the lifestyle of the participants is not directly related to the identification of motionless activities, but it is included for other analysis with this dataset.

### Data Acquisition

The data was acquired from the sensors, *i.e*., accelerometer, magnetometer, gyroscope, and GPS sensors, available in a BQ Aquaris 5.7 smartphone^12^ with a mobile application. The mobile device has a Quad Core CPU and 16 GB of internal memory. The mobile device during the data acquisition was placed at different locations, including the front pocket of the pants, a wristband, a bedside table, a table, inside the car, or different furniture. The mobile device automatically acquires the sensors’ data related to the different activities without motion, and the user selects the activity performed in the mobile application. During the data collection, also the data from the inertial sensors capture was acquired^17,18^.

**Fig. 2:**
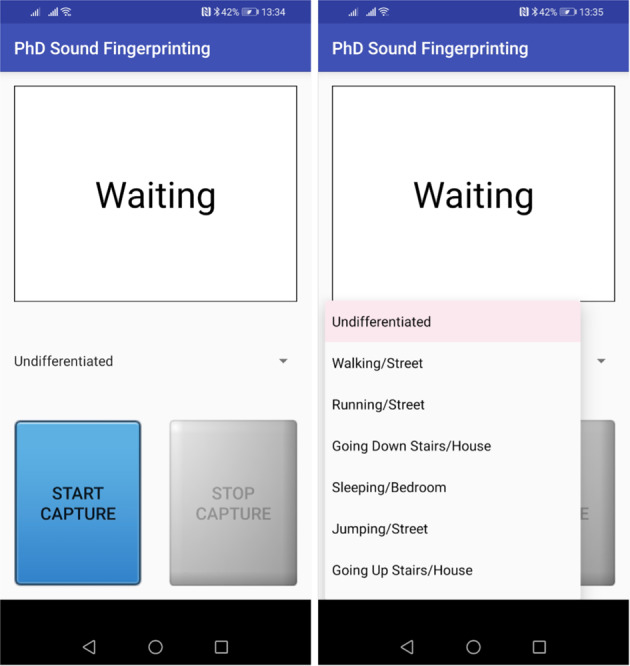
Mobile Application.

The mobile application, as presented in Fig. 2, presents a dropdown menu that allows the user to select the performed activity from a list of predefined activities. Similarly, the user also can pick the environment where the activity is occurring. It must be done before the data acquisition starts, so that the data can be labelled with the correct category. Likewise, the user also needs to insert information related to the start time, user identifier, lifestyle, age, device placement, and geographic location. The mobile application enables the capture of the accelerometer, gyroscope, magnetometer, microphone, and GPS sensors, and it stores the data in readable text files for further analysis. Each file includes 5 sec of data captured every 5 min of the use of the mobile application in the capturing stage. The source code of the mobile application is available at https://github.com/impires/DataAcquisitionADL.

Table 1 shows the different environments and the suitable mobile device placements (positions) in that environment. The procedure for data acquisition with the mobile application was explained to each participant before starting the data acquisition.

**Table 1:** Position of the smartphone during different motionless activities.

Environments	Placement
Sleeping	Over a table; Over the bedside table; Over other furniture.
Driving	Pants Pocket; On a wristband; Inside the car.
Watching TV	Over a table; Pants Pocket; On a wristband; Over other furniture.

After the preparation, the user places the mobile device in a position that she/he chooses, including the front pocket of the pants, a wristband, a bedside table, a table, inside the car, or other furniture. During the data collection, the five sensors, *i.e*., accelerometer, magnetometer, gyroscope, microphone, and Global Positioning System (GPS) sensors, collected the data at the same time, and the mobile application store it in text files for further analysis. The accelerometer, magnetometer, and gyroscope sensors are tri-axial sensors with the variables X, Y, and Z. The accelerometer has the model LIS3DHTR with a range between 0 and 32 m/s^2^, a resolution of 0.004, and a power of 0.13 mA. Next, the magnetometer or Magnetic Field sensor has a model of AKM8963C with a range between 0 and 600 m/s^2^, a resolution of 0.002, and a power of 0.25 mA. Finally, the gyroscope sensor was corrected by Google Inc, and it has a range between 0 and 34.91 m/s^2^, a resolution of 0.011, and a power of 6.48 mA. The GPS receiver has the BCM4774 Location Hub chip that integrates an advanced dual frequency GNSS receiver with a 28 nm CMOS dual processor, reporting frequencies between 10.23 MHz for GPS L5, and 1.023 MHz for GPS L1. The microphone data is collected as a byte array and stored in text files for further analysis during the data acquisition. The microphone acquires the data with a sample rate of 44100 Hz in a mono channel as an array of 16-bit unsigned integer values in the range [0, 255] with a 128 offset for zero.

The data related to the different sensors is stored so that each row is labelled with the corresponding Unix timestamp when the data was captured. The Unix timestamp denotes the time between 1^st^ of January 1970, and the current date and time in milliseconds. As the data of the different sensors can be processed independently, the synchronization problem is not relevant for the proposed purpose because data from all sensors are captured on the same mobile device, hence they have timestamps from the same clock. In a multi-device scenario, the synchronization would require additional synchronization protocols.

### Procedure

During the motionless activities, the sensors’ data were recorded with an Android application. Initially, the person selected the motionless activity that will perform in the mobile application. After that, the user pressed the start button to enable the data acquisition.

As previously mentioned, the placement of the mobile device is not fixed, rather multiple positions can be used (see Table 1). The procedure for data collection using the mobile application was explained to each participant and consists of the following steps:

1. Install the mobile application on the mobile device;
2. Open the mobile application designed for the acquisition of the sensors’ data;
3. The user selects the motionless activity that he/she will perform;
4. Press the button to start the data acquisition;
5. The data acquisition starts after 10 sec;
6. The user positions the mobile device adequately;
7. The data acquisition is performed during slots of 5 sec;
8. The data acquisition stops for 5 min;
9. The flow returns to point 7, and it repeats continuously until the user press the stop button.

## Data Records

The dataset presented in this paper is available in a Mendeley Data repository^19^, and it contains three main folders, *i.e*., one folder for each motionless activity. Each one of the three folders contains more than 2000 numbered folders with the files related to the data acquired from the various sensors. Thus, each subfolder contains five files named as “accelerometer.txt”, “magnetometer.txt”, “gyroscope.txt”, “location.txt”, and “sound.txt”. In total the dataset contains around 6000 files for each sensor. Regarding the files related to accelerometer, magnetometer, and gyroscope sensors, the values are collected in m/s^2^. On the other way, the files related to GPS received contains to columns with the geographical coordinates, including latitude, and longitude. Finally, the acoustic data contained the byte arrays, where each value is presented in only one column.

The following columns are presented in the files related to the accelerometer data:

- First column: Timestamp of each sample (ms);
- Second column: Value of the x-axis of the accelerometer (m/s^2^);
- Third column: Value of the y-axis of the accelerometer (m/s^2^);
- Fourth column: Value of the z-axis of the accelerometer (m/s^2^).

The following columns are presented in the files related to the magnetometer sensor:

- First column: Timestamp of each sample (ms);
- Second column: Value of the x-axis of the magnetometer (m/s^2^);
- Third column: Value of the y-axis of the magnetometer (m/s^2^);
- Fourth column: Value of the z-axis of the magnetometer (m/s^2^).

The following columns are presented in the files related to the gyroscope sensor:

- First column: Timestamp of each sample (ms);
- Second column: Value of the x-axis of the gyroscope (m/s^2^);
- Third column: Value of the y-axis of the gyroscope (m/s^2^);
- Fourth column: Value of the z-axis of the gyroscope (m/s^2^).

The following columns are presented in the files related to the GPS sensor:

- First column: Timestamp of each sample (ms);
- Second column: Value of the latitude;
- Third column: Value of the longitude.

The following column is presented in the files related to acoustic data:

- First column: Integer value related to the byte arrays collected from the microphone.

**Fig. 3:**
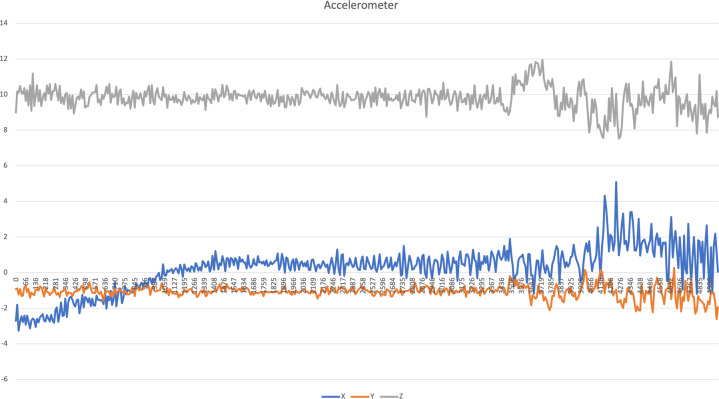
Accelerometer data related to driving activity.

**Fig. 4:**
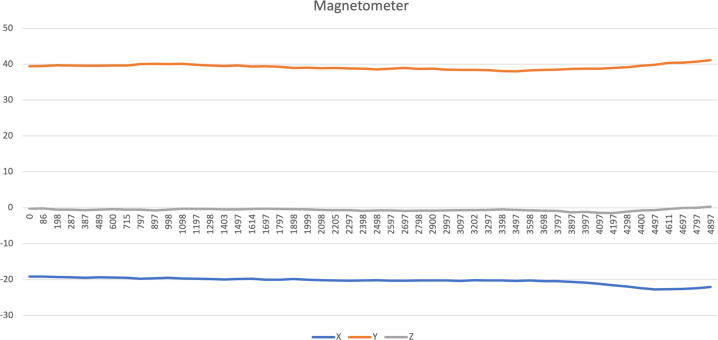
Magnetometer data related to driving activity.

**Fig. 5:**
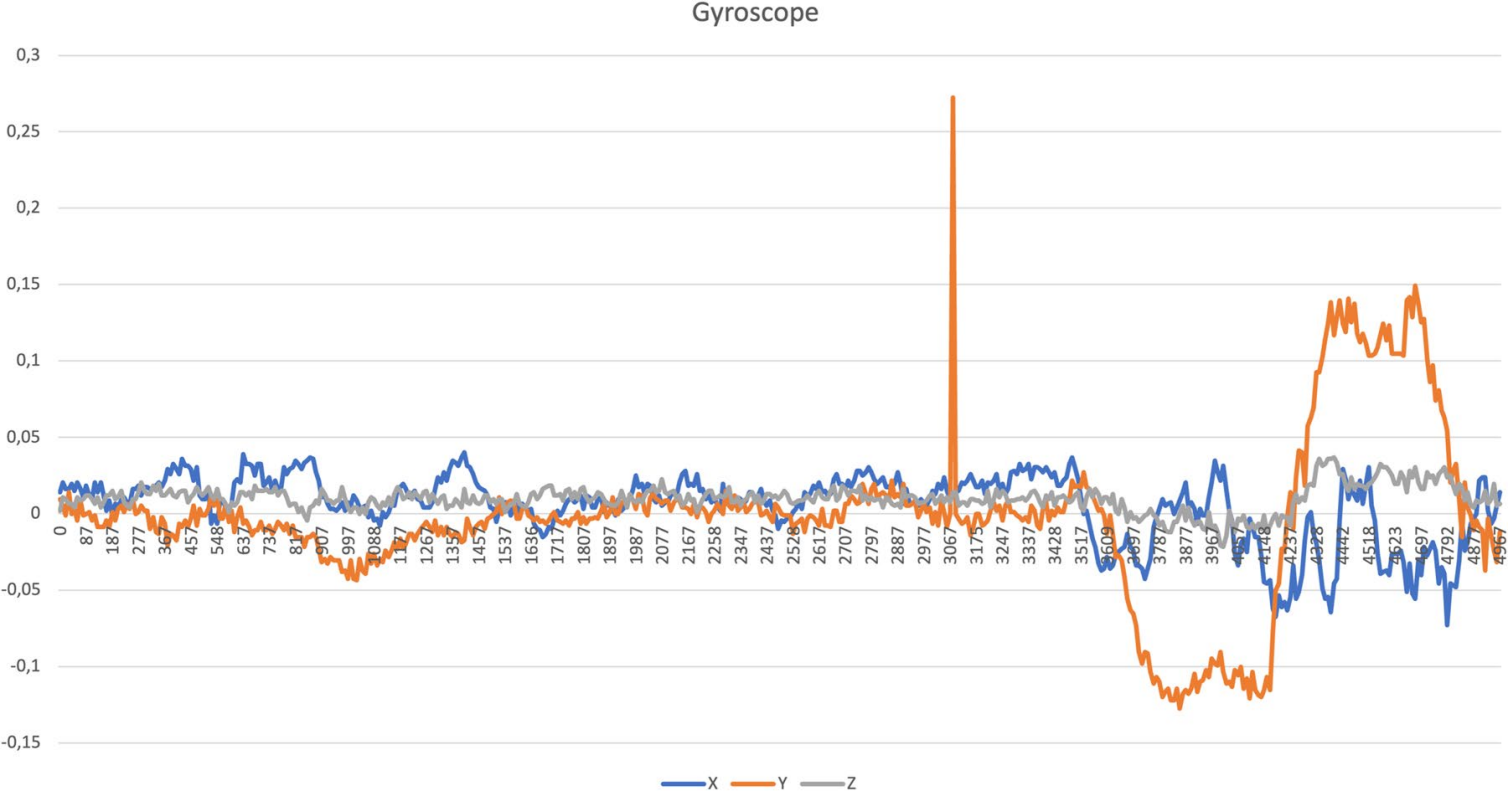
Gyroscope data related to driving activity.

**Fig. 6:**
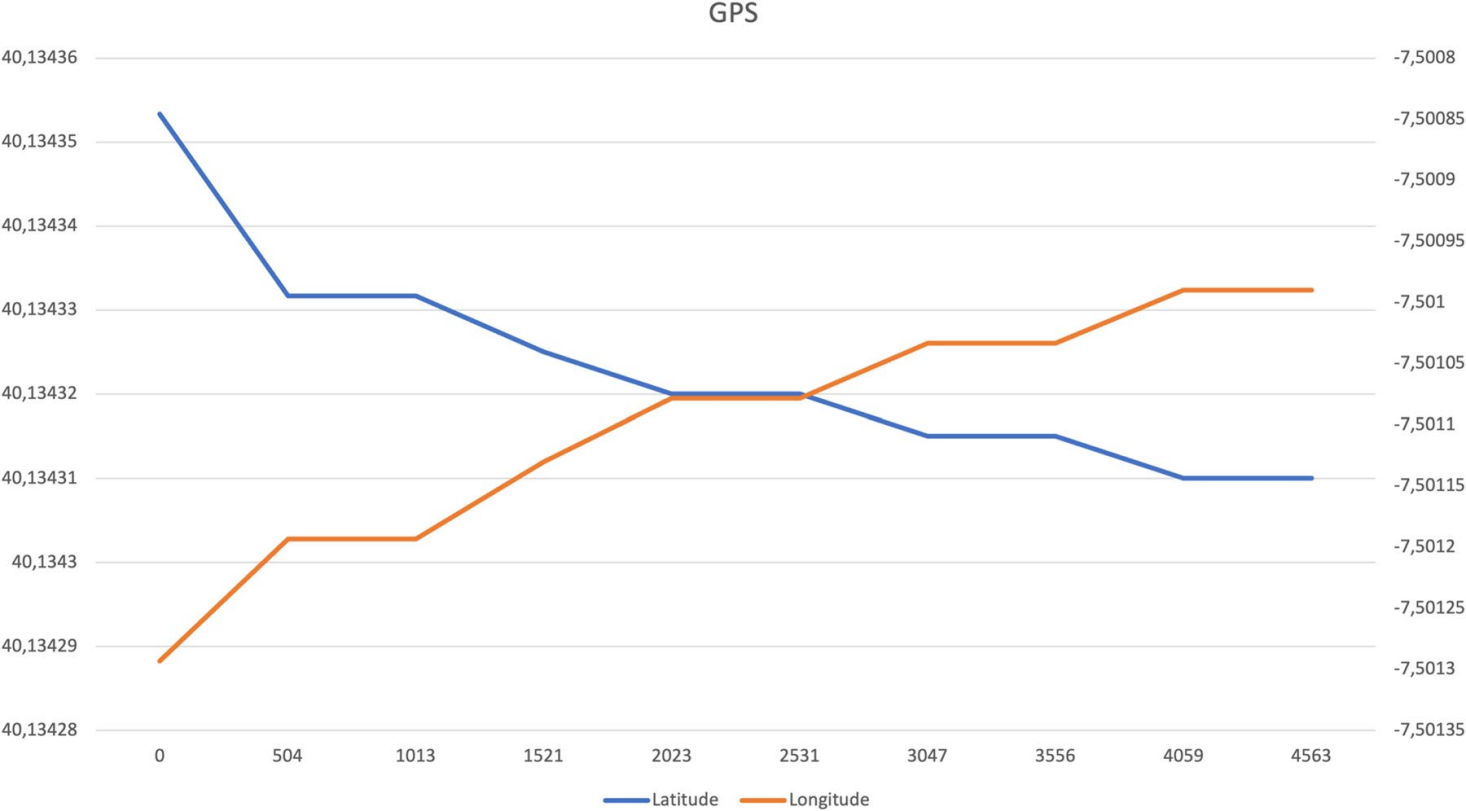
GPS data related to driving activity.

**Fig. 7:**
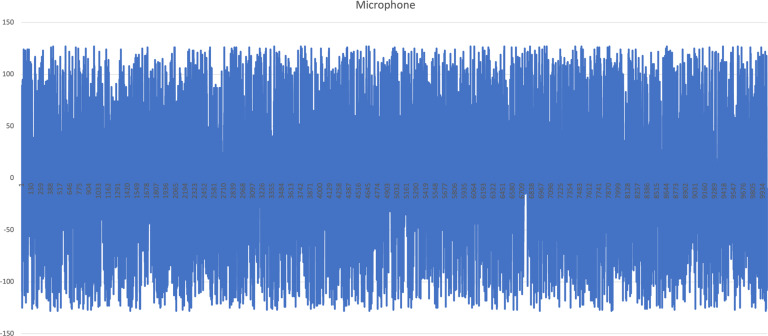
Excerpt of 10000 samples of microphone data related to driving activity as byte array.

The charts related to driving activity are presented in Figs. 3–7 to illustrate the acquired data. The accelerometer, magnetometer, gyroscope, and GPS data include the whole 5 sec of data. The presented acoustic data that is visualized in Fig. 7 is an excerpt with 10000 samples. However, the original files for all sensors are available in the following links:

- Accelerometer data:
  - https://data.mendeley.com/datasets/3dc7n482rt/3/files/7285df73-ef14-4855-823d-36585f8cfcf5
- Magnetometer data:
  - https://data.mendeley.com/datasets/3dc7n482rt/3/files/b76547be-3526-4cf8-974e-fd1041e4bdb2
- Gyroscope data:
  - https://data.mendeley.com/datasets/3dc7n482rt/3/files/09aa6e8b-23cd-4116-983f-22952f4a0310
- GPS data:
  - https://data.mendeley.com/datasets/3dc7n482rt/3/files/dbf8972f-fa4d-4d1f-bec3-6c1dfa57ce86
- Microphone data:
  - https://data.mendeley.com/datasets/3dc7n482rt/3/files/84c43dde-acc8-42a5-9966-6abdf3af859f

Considering the environments recognized by the framework presented in previous studies^18,20^, we considered the environments presented in Table 2 for the analysis of the data from the different folders.

**Table 2:** Environments for the acquired data.

Activity	Environment
Sleeping	Bedroom
Driving	Street
Watching TV	Living room

Thus, different combinations of sensors are performed. These are:

- Accelerometer + Environment + GPS;
- Accelerometer + Magnetometer + Environment + GPS;
- Accelerometer + Magnetometer + Gyroscope + Environment + GPS.

For each inertial sensor, *i.e*., accelerometer, magnetometer, and gyroscope, the Euclidean norm^21^ was measured for each row of the different files. It was used for the measurement of a set of features for further analysis of each sensor, as presented in^17^.

For the acoustic data, the Mel-frequency cepstral coefficients (MFCC)^22^ were measured for each file. It was used for the measurement of a set of features for further analysis previously defined^18^.

For the GPS data, the distance (in meters) along the data available in each file was measured, and it was used as the unique feature extracted from the GPS data^20^.

The source code used for the measurement of the different features is available at https://github.com/impires/FeatureExtractionMotionlessActivities.

Table 3 presents the average of the different measured parameters of all Accelerometer + Environment + GPS samples of the data acquisition related to each motionless activity.

**Table 3:** Average of the parameters calculated for each motionless activity with Accelerometer + Environment + GPS samples.

Sensor	Parameters	Sleeping	Driving	Watching TV
Accelerometer	Average distance between five highest peaks (ms)	504.87	491.77	518.66
	Average of maximum peaks (m/s^2^)	9.73	10.22	9.81
	Standard deviation of maximum peaks (m/s^2^)	0.01	0.39	0
	Variance of maximum peaks (m/s^2^)	0	0.20	0
	Median of maximum peaks (m/s^2^)	9.72	10.19	9.81
	Average of raw data (m/s^2^)	0.02	0.44	0.01
	Standard deviation of raw data (m/s^2^)	9.70	9.68	9.79
	Maximum of raw data (m/s^2^)	9.76	11.10	9.83
	Minimum of raw data (m/s^2^)	9.65	8.38	9.76
	Variance of raw data (m/s^2^)	0	0.23	0
	Median of raw data (m/s^2^)	9.71	9.90	9.80
GPS	Distance (m)	2.05	111.18	3.77

Table 4 presents the average of the different measured parameters of all Accelerometer + Magnetometer + Environment + GPS samples of the data acquisition related to each motionless activity.

**Table 4:** Average of the parameters calculated for each motionless activity with Accelerometer + Magnetometer + Environment + GPS samples.

Sensor	Parameters	Sleeping	Driving	Watching TV
Accelerometer	Average distance between five highest peaks (ms)	504.87	491.77	502.71
	Average of maximum peaks (m/s^2^)	9.73	10.22	10.22
	Standard deviation of maximum peaks (m/s^2^)	0.01	0.39	0.39
	Variance of maximum peaks (m/s^2^)	0	0.20	0.20
	Median of maximum peaks (m/s^2^)	9.72	10.19	10.19
	Average of raw data (m/s^2^)	0.02	0.44	0.44
	Standard deviation of raw data (m/s^2^)	9.70	9.68	9.68
	Maximum of raw data (m/s^2^)	9.76	11.10	11.10
	Minimum of raw data (m/s^2^)	9.65	8.38	8.38
	Variance of raw data (m/s^2^)	0	0.23	0.23
	Median of raw data (m/s^2^)	9.71	9.90	9.90
Magnetometer	Average distance between five highest peaks (ms)	135.36	139.60	139.60
	Average of maximum peaks (m/s^2^)	42.56	29.48	29.48
	Standard deviation of maximum peaks (m/s^2^)	0.28	0.70	0.70
	Variance of maximum peaks (m/s^2^)	0.10	1.49	1.49
	Median of maximum peaks (m/s^2^)	42.58	29.51	29.51
	Average of raw data (m/s^2^)	0.28	0.70	0.70
	Standard deviation of raw data (m/s^2^)	42.56	29.48	29.48
	Maximum of raw data (m/s^2^)	43.01	30.65	30.65
	Minimum of raw data (m/s^2^)	41.97	28.27	28.27
	Variance of raw data (m/s^2^)	0.10	1.49	1.49
	Median of raw data (m/s^2^)	42.58	29.49	29.49
GPS	Distance	2.05	111.18	111.18

Table 5 presents the average of the different measured parameters of all Accelerometer + Magnetometer + Gyroscope + Environment + GPS samples of the data acquisition related to each motionless activity.

**Table 5:** Average of the parameters calculated for each motionless activity with Accelerometer + Magnetometer + Gyroscope + Environment + GPS samples.

Sensor	Parameters	Sleeping	Driving	Watching TV
Accelerometer	Average distance between five highest peaks (ms)	506.38	491.77	518.66
	Average of maximum peaks (m/s^2^)	9.92	10.22	9.81
	Standard deviation of maximum peaks (m/s^2^)	0.14	0.39	0.01
	Variance of maximum peaks (m/s^2^)	0.07	0.20	0
	Median of maximum peaks (m/s^2^)	9.91	10.19	9.81
	Average of raw data (m/s^2^)	0.16	0.44	0.01
	Standard deviation of raw data (m/s^2^)	9.73	9.68	9.79
	Maximum of raw data (m/s^2^)	10.23	11.10	9.83
	Minimum of raw data (m/s^2^)	9.26	8.38	9.76
	Variance of raw data (m/s^2^)	0.08	0.23	0
	Median of raw data (m/s^2^)	9.80	9.90	9.80
Magnetometer	Average distance between five highest peaks (ms)	138.73	139.60	141.22
	Average of maximum peaks (m/s^2^)	36.30	29.48	36.85
	Standard deviation of maximum peaks (m/s^2^)	0.41	0.70	0.25
	Variance of maximum peaks (m/s^2^)	0.55	1.49	0.08
	Median of maximum peaks (m/s^2^)	36.32	29.51	36.87
	Average of raw data (m/s^2^)	0.41	0.70	0.26
	Standard deviation of raw data (m/s^2^)	36.30	29.48	36.85
	Maximum of raw data (m/s^2^)	36.98	30.65	37.27
	Minimum of raw data (m/s^2^)	35.53	28.27	36.34
	Variance of raw data (m/s^2^)	0.56	1.49	0.08
	Median of raw data (m/s^2^)	36.31	29.49	36.86
Gyroscope	Average distance between five highest peaks (ms)	457.99	418.74	488.90
	Average of maximum peaks (m/s^2^)	0.04	0.06	0.03
	Standard deviation of maximum peaks (m/s^2^)	0.02	0.02	0.01
	Variance of maximum peaks (m/s^2^)	0	0	0
	Median of maximum peaks (m/s^2^)	0.03	0.06	0.02
	Average of raw data (m/s^2^)	0.01	0.02	0
	Standard deviation of raw data (m/s^2^)	0.03	0.05	0.02
	Maximum of raw data (m/s^2^)	0.09	0.12	0.07
	Minimum of raw data (m/s^2^)	0.02	0.02	0.02
	Variance of raw data (m/s^2^)	0	0	0
	Median of raw data (m/s^2^)	0.03	0.05	0.02
GPS	Distance	39.00	111-18	3.77

Tables 3, 4, and 5 clearly show that the minimum, maximum, average, standard deviation, variance and median of the different sensory data is different between the various activities. Of course, with different machine learning algorithms this raw data can be processed so that complex relationships between the data are better understood. But even these aggregate descriptive statistics indicate that such trained models could differentiate the different activities and environments.

The aggregate data presented in Tables 3, 4, and 5 was computed with a Java program based on the raw data. Additionally, Python and Jupyter were used for the data exploration. All code used for that is provided, as detailed in the Code Availability section.

### Missing data information

The missing data corresponds to the number of missing values available based on the identification of the frequency of the data acquisition. Its identification started with the analysis of the number of samples needed for the whole 5 sec by sensor. The frequency rate for accelerometer and gyroscope sensors was 100 Hz (*i.e*., 100 samples/s), while for magnetometer, it was 10 Hz (*i.e*., 10 samples/s). Regarding the GPS received, the frequency rate corresponds to 2 Hz (*i.e*., 2 samples/s). For each capture, there should be 5 × 100 = 500 samples for the accelerometer and gyroscope sensors, 5 × 10 = 50 samples for the magnetometer sensor, and 5 × 2 = 10 samples for GPS receiver values. For some instances for Watching TV and Sleeping activities, the GPS sensor values are not present. Regarding the microphone data, we only collected the audio data as a byte array, and the data imputation is not needed for the classification. Table 6 shows the analysis of the missing samples in the provided dataset, categorizing it by at least 90% of fulfilled data, *i.e*., 450 samples for accelerometer and gyroscope sensors, 45 samples for magnetometer sensor, and 9 samples for GPS receiver values, at least 80% of fulfilled data, *i.e*., 400 samples for accelerometer and gyroscope sensors, 40 samples for magnetometer sensor, and 8 samples for GPS receiver values, and less than 80% of fulfilled data.

**Table 6:** Number of valid or non-valid samples.

Activity	Sensors	Total Number of Samples	Number of Samples Fulfilled	Number of Samples 90% Fulfilled	Number of Samples 80% Fulfilled	Number of Samples < 80% Fulfilled
Sleeping	Accelerometer	2207	0	2145	2198	9
	Magnetometer	2207	1481	2207	2207	0
	Gyroscope	2206	1	2144	2193	13
	GPS receiver	1586	1289	1586	1586	0
Driving	Accelerometer	2161	27	2095	2098	63
	Magnetometer	2161	1580	2098	2101	60
	Gyroscope	2161	3	2098	2101	60
	GPS receiver	2025	1669	2023	2025	0
Watching TV	Accelerometer	1747	7	1743	1745	2
	Magnetometer	1747	1140	1744	1745	2
	Gyroscope	1747	3	1743	1745	2
	GPS receiver	940	788	939	939	1

The analysis of the missing data allowed to verify that most of the data is useful for the correct classification with at least 90% of data, where a major part of the data is reliable for the correct identification. Regarding the sleeping activity, 97% of the data acquired from the accelerometer and gyroscope sensors are reliable, and 100% of the data acquired from the magnetometer and GPS receiver is reliable. Regarding the driving activity, 97% of the acquired from the accelerometer, magnetometer and gyroscope sensors are reliable, and 99% of the data acquired from the GPS receiver is reliable. Finally, regarding the watching TV activity, 99% of the acquired from all sensors is reliable.

## Technical Validation

The quality of the data is important for the correct recognition of the activities of daily living and environments. Initially, we started with the validation of the availability of the whole 5 sec of data on each dataset. We revealed that records have incomplete data, so they should either be discarded, or data imputation techniques must be applied to fix these data inconsistencies.

For the validation of the acquired data, different machine learning methods were tested, including k-Nearest Neighbors, Linear SVM, RBF SVM, Decision Tree, Random Forest, Neural Networks, AdaBoost, Naive Bayes, QDA, and XGBoost. The configurations of the different methods are detailed in Jupyter notebook (https://github.com/impires/JupyterNotebooksMotionlessActivities).

After the implementation of the different methods, the reported results are presented in Table 7.

**Table 7:** Classification details.

Classifier	Accuracy	Precision	Recall	F1-Score
k-Nearest Neighbors	100%	100%	99%	99%
Linear SVM	100%	100%	99%	99%
RBF SVM	100%	100%	99%	99%
Decision Tree	100%	100%	99%	99%
Random Forest	100%	100%	99%	99%
Neural Networks	100%	100%	99%	99%
AdaBoost	100%	100%	99%	99%
Naive Bayes	100%	100%	99%	99%
QDA	100%	100%	99%	99%
XGBoost	100%	100%	99%	99%

## Usage Notes

The potential applications of this dataset range are related to activity recognition. Unlike most datasets publicly available for this purpose, this dataset also allows considering the context (i.e., environment) where the activity is happening. Additionally, providing the microphone data can inspire other uses of the dataset related to ambient assisted living. In such cases, the audio data can provide important validation of the recognized activities. For example, lying in the living room with the TV on (detectable with the audio sensor) is not concerning. However, lying in the bathroom is a safety concern. So just identifying the activity (lying) is not sufficient and can mean different things in different contexts. This dataset can initiate such research. However, the limitations are related to the dataset size and the privacy concerns that the audio data raises. In this dataset, this concern is already addressed by the data collection protocol and participants consent, but in general, data collection should pay close attention to such concerns.

## Data Availability

The Android project related to the mobile application used for the data acquisition from all sensors is available at https://github.com/impires/DataAcquisitionADL. In addition, the Java project used for the automatic measurement of the parameters of related to the different sensors is available at https://github.com/impires/FeatureExtractionMotionlessActivities. The code for preliminary data exploration and analysis is available as a Jupyter notebook at https://github.com/impires/JupyterNotebooksMotionlessActivities. The Jupyter notebook shows how the data can be loaded, and how the initial data exploration can be performed showing some charts and descriptive statistics. This will be more than sufficient to bootstrap future uses of the dataset.
